# Decreased STAT3 Phosphorylation Mediates Cell Swelling in Ammonia-Treated Astrocyte Cultures

**DOI:** 10.3390/biology5040048

**Published:** 2016-12-02

**Authors:** Arumugam R. Jayakumar, Kevin M. Curtis, Kiran S. Panickar, Nagarajarao Shamaladevi, Michael D. Norenberg

**Affiliations:** 1 Department of Pathology, Miller School of Medicine, University of Miami, Miami, FL 33101, USA; kiran_panickar@hillspet.com; 2 South Florida Foundation for Research and Education Inc., Miami VA Healthcare System, Miami, FL 33125, USA; nithura7@yahoo.com; 3 Veterans Affairs Medical Center, U.S. Department of Veterans Affairs, Miami, FL 33125, USA; 4 Department of Biochemistry & Molecular Biology, Miller School of Medicine, University of Miami, Miami, FL 33101, USA; kevinmcurtis@gmail.com; 5 Geriatric Research Education and Clinical Center, U.S. Department of Veterans Affairs, Miami, FL 33125, USA; 6 Department of Urology, Miller School of Medicine, University of Miami, Miami, FL 33101, USA

**Keywords:** acute liver failure, ammonia, astrocyte swelling, STAT3 dephosphorylation, protein tyrosine phosphatase receptor type-1

## Abstract

Brain edema, due largely to astrocyte swelling, and the subsequent increase in intracranial pressure and brain herniation, are major complications of acute liver failure (ALF). Elevated level of brain ammonia has been strongly implicated in the development of astrocyte swelling associated with ALF. The means by which ammonia brings about astrocyte swelling, however, is incompletely understood. Recently, oxidative/nitrosative stress and associated signaling events, including activation of mitogen-activated protein kinases (MAPKs), as well as activation of the transcription factor, nuclear factor-kappaB (NF-κB), have been implicated in the mechanism of ammonia-induced astrocyte swelling. Since these signaling events are known to be regulated by the transcription factor, signal transducer and activator of transcription 3 (STAT3), we examined the state of STAT3 activation in ammonia-treated cultured astrocytes, and determined whether altered STAT3 activation and/or protein expression contribute to the ammonia-induced astrocyte swelling. STAT3 was found to be dephosphorylated (inactivated) at Tyrosine705 in ammonia-treated cultured astrocytes. Total STAT3 protein level was also reduced in ammonia-treated astrocytes. We also found a significant increase in protein tyrosine phosphatase receptor type-1 (PTPRT-1) protein expression in ammonia-treated cultured astrocytes, and that inhibition of PTPRT-1 enhanced the phosphorylation of STAT3 after ammonia treatment. Additionally, exposure of cultured astrocytes to inhibitors of protein tyrosine phosphatases diminished the ammonia-induced cell swelling, while cultured astrocytes over-expressing STAT3 showed a reduction in the astrocyte swelling induced by ammonia. Collectively, these studies strongly suggest that inactivation of STAT3 represents a critical event in the mechanism of the astrocyte swelling associated with acute liver failure.

## 1. Introduction

Brain edema, due largely to astrocyte swelling, and the subsequent increase in intracranial pressure and brain herniation, are major complications of acute liver failure (ALF). Elevated levels of brain ammonia have been strongly implicated in the development of the astrocyte swelling (cytotoxic brain edema) associated with ALF [1,2,3]. The mechanism by which ammonia causes astrocyte swelling in ALF, however, is poorly understood. Oxidative/nitrosative stress (ONS) and associated signaling events, including the activation of mitogen-activated protein kinases (MAPKs), as well as transcription factors p53, and nuclear factor-kappaB (NF-κB), have been implicated in the mechanism of ammonia-induced astrocyte swelling [4,5,6,7]. Of relevance, all of these signaling molecules are known to be regulated by the transcription factor, signal transducer and activator of transcription 3 (STAT3).

STAT3 is a transcription factor which in humans is encoded by the STAT3 gene. Constitutive STAT3 activation is associated with various neoplasms that have anti-apoptotic and proliferative effects [8,9,10,11,12,13]. Activation of STAT3 was shown to protect hepatocytes from hypoxic/reoxygenation-induced oxidative stress and subsequent cell injury by the upregulation of the antioxidant enzyme manganese superoxide dismutase in vitro [14] and in vivo [15]. Alternatively, loss-of-function by mutations in the STAT3 gene results in recurrent infections, as well as disordered bone and tooth development. STAT3 deletion was shown also to promote oxidative stress in cardiomyocytes [16], and to activate p53 in leukemic cells [17], whose activation was subsequently shown to induce cell swelling in ammonia-treated astrocytes [6]. Additionally, Yu et al. [18] reported that increased phosphorylation (activation) of STAT3 inhibited the activation of NF-κB as well as the inducible nitric oxide synthase gene expression, factors well-known to be involved in the mechanisms of cell swelling [4,5]. Additionally, Sinn et al. [19] observed that phosphorylated STAT3 was decreased in brain following intracerebral hemorrhage in rats, a neurological condition also associated with the development of brain edema. Together, these findings suggest that dephosphorylation of STAT3 in astrocytes in ALF may represent an important factor in the mechanism of astrocytic cell volume regulation. This study therefore investigated the state of STAT3 activation in ammonia-treated cultured astrocytes, and its potential involvement in the mechanism of the astrocyte swelling associated with elevated levels of ammonia.

## 2. Experimental Procedures

### 2.1. Astrocyte Cultures

Cultured cortical astrocytes were prepared as described previously [20]. Briefly, brains of 1–2 day old rat pups were seeded on 35 mm plastic dishes in Dulbecco’s Modified Eagle Medium containing 15% fetal bovine serum (FBS), penicillin and streptomycin. The culture plates were then incubated with 5% CO_2_ and 95% air at 37 °C. Culture media were changed twice weekly. On Day 10 post seeding, FBS was replaced with 10% horse serum. On Day 14, cultures were treated with dibutyryl cyclic AMP (db-cAMP) (0.5 mM) to enhance cellular differentiation [21]. Cultures consisted of 95%–99% astrocytes as determined by glial fibrillary acidic protein (GFAP) immunocytochemistry. All cultures used in the experiments were 25–28 days old. Procedures followed guidelines established by National Institute of Health *Guide for the Care and use of Laboratory Animals* and were approved by the local Animal Care and Use Committee (IACUC).

### 2.2. Cell Volume Determination

Cell volume was determined by measuring the intracellular water space using the method of Kletzien et al. [22], as modified by Kimelberg [23] and Bender and Norenberg [24]. Briefly, 1 mM 3-*O*-methylglucose (3-OMG) and 0.5 µCi/mL [3H]-3-OMG were added to the cultures 6 h before the volume assay. At the end of the incubation period, culture media were aspirated, and aliquots were saved for radioactivity determination. Cells were washed five-six times with ice-cold buffer (229 mM sucrose, 1 mM Tris-nitrate, 0.5 mM calcium nitrate, and 0.1 mM phloretin, pH 7.4) and lysed with 0.5 mL of 1 M sodium hydroxide. Radioactivity in the cell extracts and medium were measured using liquid scintillation counter. An aliquot of the cell extract was then used for protein estimation using the BCA kit, and the values were normalized to protein level. Cell volume was expressed as microliters/milligram protein.

### 2.3. Overexpression of STAT3

To examine whether the overexpression of STAT3 can diminish/prevent the ammonia-induced STAT3 dephosphorylation, as well as cell swelling, we overexpressed STAT3 in cultured astrocytes as previously described [25]. Briefly, the pExpress-1 mammalian overexpression vector (Open Biosystems) containing the rat STAT3 ORF clone (Accession# BC087025) was used to overexpress STAT3 in cultured astrocytes. Mirus TransIT-TKO transfection reagent was used to transfect STAT3 following the manufacturer guidelines (Mirus: #MIR 2150). Astrocyte cultures were exposed to different concentrations of STAT3 cDNA (50, 100 and 200 nm) for 24 h, 48 h and 72 h, and at the end of transfection, media from these cultures were aspirated, cells washed with fresh media and maintained in fresh media for 12–24 h. The empty pExpress-1 vector and the transfection reagent alone were used as controls for all experiments. To determine the extent of STAT3 overexpression in astrocytes, both total and phosphorylated STAT3 protein expression in control and STAT3 overexpressed cells were measured by Western blots.

### 2.4. Western Blots

Control cells, or cells treated with ammonia, were subjected to gel electrophoresis and immunoblotting as described previously [4]. An antibody against p-STAT3Tyr705 (D3A7) XP^TM^ rabbit mAb (cat#9145, Cell Signaling Technology, Beverly, MA, USA, which did not cross-react with EGFR and α-tubulin) was used at 1:1000 dilutions. Primary antibody to protein tyrosine phosphatase receptor type-1 (PTP1B Antibody or PTPRT-1, cat# PA1-27631) was purchased from Thermo Fisher Scientific (Waltham, MA, USA), and was used at 1:1000 dilution. Horseradish peroxidase-conjugated anti-rabbit secondary antibody (1:1000) (Vector Laboratories, Burlingame, CA, USA) was used. Optical density of the bands was measured with the Chemi-Imager digital imaging system (Alpha Innotech, San Leandro, CA, USA), and the results were quantified with the Sigma Scan Pro program (Jandell Scientific, San Jose, CA, USA) as a proportion of the signal of a house-keeping protein band (α-tubulin) [4,25].

### 2.5. Statistical Analysis

Each experimental group consisted of four to five culture dishes for each time point examined for the determination of cell swelling. Western blot (WB) analyses were performed from two to four cell culture plates from four to seven separate seedings. Cell swelling values were normalized to protein values and analyzed by ANOVA (StatMost, Dataxiom Software Inc., Los Angeles, CA, USA) followed by Tukey’s post hoc comparisons. WB band intensity unit values were also subjected to ANOVA followed by Tukey’s post hoc comparison test, and these values were expressed as a percentage change over control. At each time point, the experimental cultures were compared with their respective control.

## 3. Results

### 3.1. Effect of Ammonia on STAT3 Phosphorylation in Cultured Astrocytes

**Figure 1:**
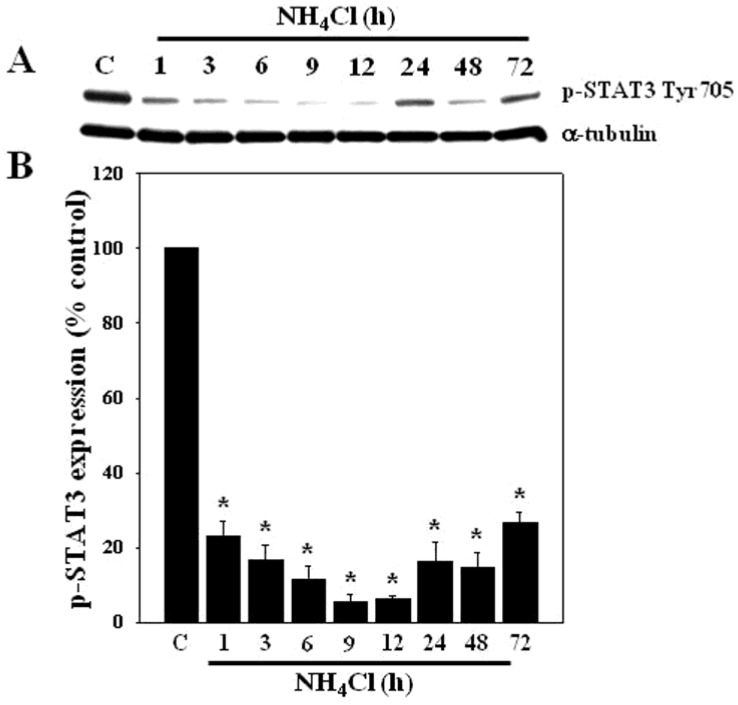
Effect of ammonia on STAT3 Tyr705 phosphorylation in cultured astrocytes. (**A**) Immunoblots of phosphorylated STAT3 Tyr705 obtained at different time points after ammonia treatment. STAT3 was dephosphorylated after ammonia treatment (NH_4_Cl; 5 mM) at all time points examined (0–72 h); (**B**) Quantification of STAT3 phosphorylation. Data were subjected to ANOVA followed by Tukey‘s post hoc comparison test (*n =* 4). * *p* < 0.05 vs. control. Error bars, mean ± S.E. C, control.

Astrocyte cultures were exposed to a pathophysiological concentration of ammonia (NH_4_Cl; 5 mM) for different time periods (1–72 h), as this concentration has been observed in brains of experimental animals with acute liver failure [26,27,28]. At the end of treatment, the extent of STAT3 phosphorylation was determined by Western blots (WB). Cultures exposed to ammonia showed a marked decline in STAT3-Tyr705 phosphorylation at all time points examined (Figure 1).

Ammonia did not alter the phosphorylation of STAT3 on the serine residue 727, nor the state of tyrosine phosphorylation of STAT1 [29] (Unpublished observation).

**Figure 2:**
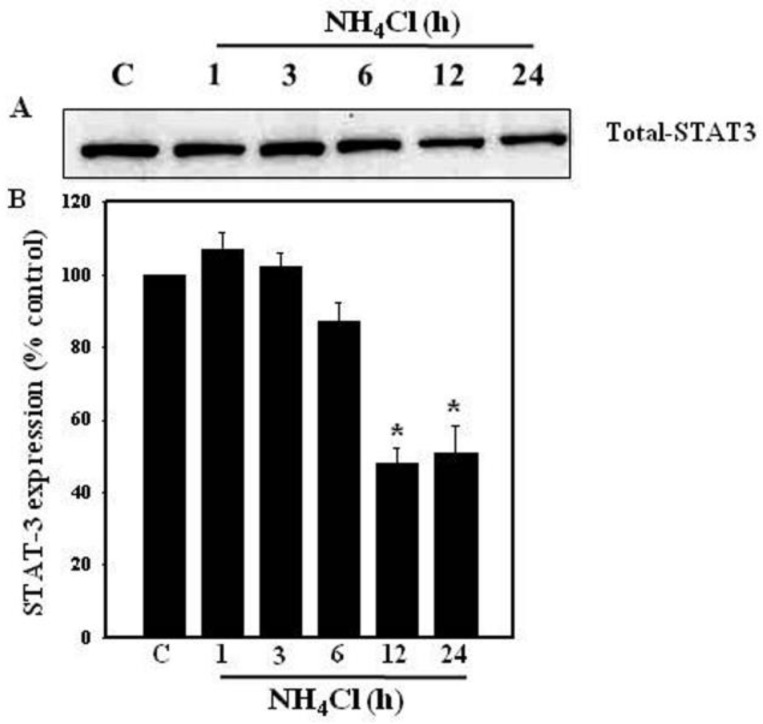
Effect of ammonia on STAT3 protein expression in cultured astrocytes. (**A**) Immunoblots of total STAT3 protein expression at different time points (0–24 h) after ammonia treatment (NH_4_Cl; 5 mM). Ammonia reduced the level of total STAT3 protein at 12 and 24 h; (**B**) Quantification of STAT3 protein expression. Data were subjected to ANOVA followed by Tukey‘s post hoc comparison test (*n =* 5). * *p* < 0.05 vs. control. Error bars, mean ± S.E. C, control.

**Figure 3:**
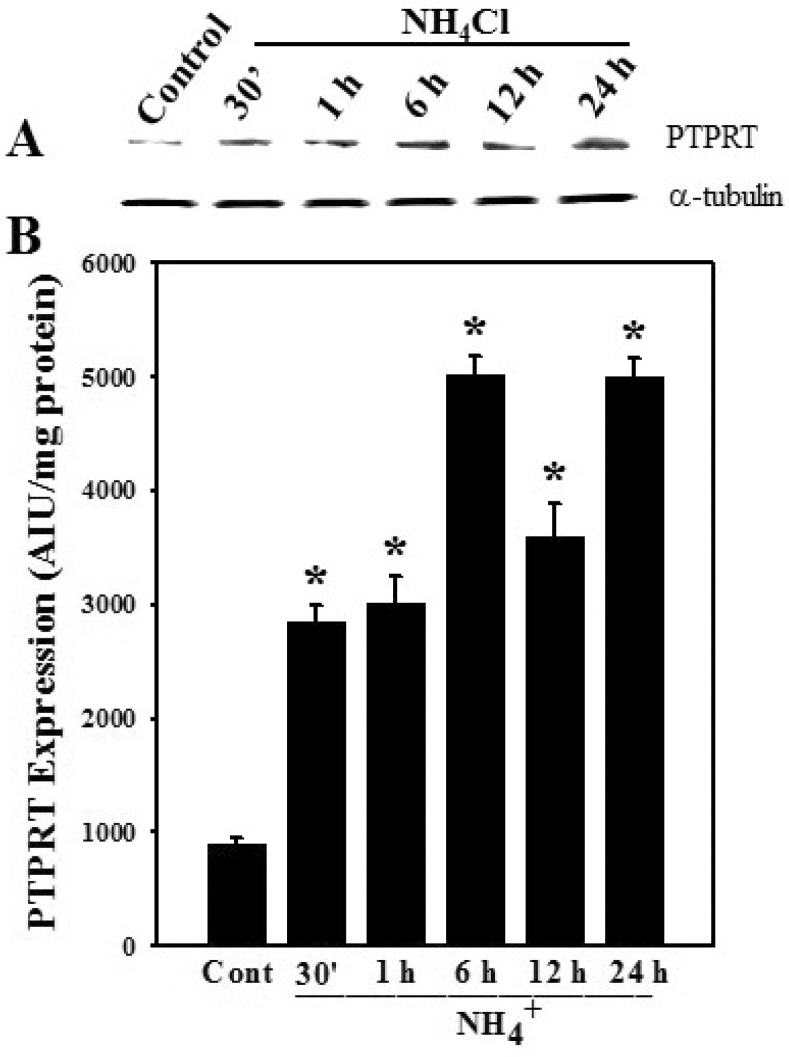
Effect of ammonia on PTPRT expression in cultured astrocytes. (**A**) Immunoblots of PTPRT at different time points (0–24 h) after ammonia treatment (NH_4_Cl; 5 mM). PTPRT expression was increased after ammonia treatment at all time points examined; (**B**) Quantification of PTPRT expression. Data were subjected to ANOVA followed by Tukey‘s post hoc comparison test (*n =* 4). * *p* < 0.05 vs. control. Error bars, mean ± S.E. Cont, control.

**Figure 4:**
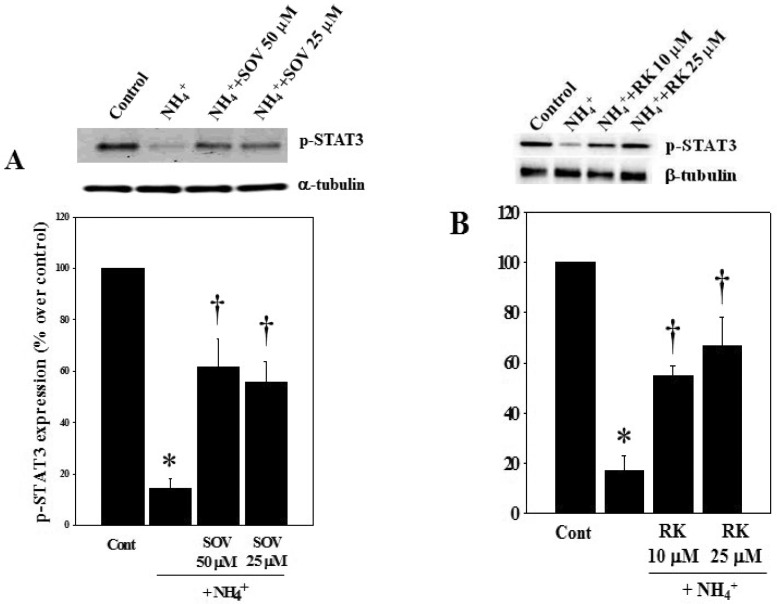
Effect of a protein phosphatase inhibition on the ammonia-induced (NH_4_Cl; 5 mM) STAT3Tyr705 phosphorylation (24 h) in cultured astrocytes. (**A**) Western blot analysis displayed a reversal of the ammonia-induced STAT3Tyr705 dephosphorylation after pretreatment (15 min) of cultures with 25 μM and 50 μM of sodium orthovanadate (SOV); (**B**) Western blot analysis displayed a reversal of the ammonia-induced STAT3Tyr705 dephosphorylation after pretreatment (15 min) of cultures with 10 μM and 25 μM of RK-682 (RK). Data were subjected to ANOVA followed by Tukey‘s post hoc comparison test (*n =* 4). * *p* < 0.05 vs. control. † *p* < 0.05 vs. Ammonia. Error bars, mean ± S.E. Cont, control. NH_4_^+^, NH_4_Cl.

**Figure 5:**
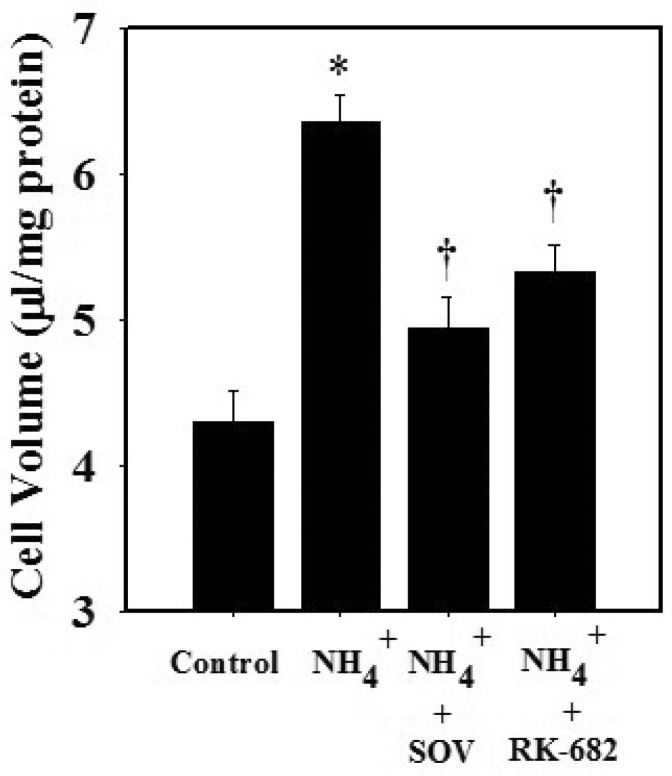
Effect of the protein tyrosine phosphatase (PTP) inhibitors (sodium orthovanadate and RK-682) on the ammonia-induced (NH_4_Cl; 5 mM) astrocyte swelling (24 h). PTP inhibitors (50 μM SOV and 25 μM RK-682) markedly reduced the cell swelling induced by ammonia. Data were subjected to ANOVA followed by Tukey‘s post hoc comparison test (*n =* 4). * *p* < 0.05 vs. control † *p* < 0.05 vs. NH_4_^+^. Error bars, mean ± S.E. OSV, sodium orthovanadate.

**Figure 6:**
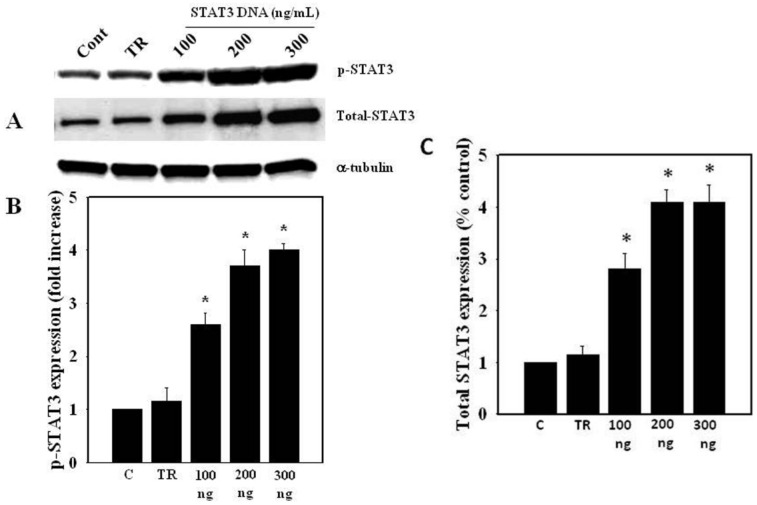
Total and phosphorylated STAT3 after astrocyte cultures were transfected with different concentrations of pExpress1-STAT3 (100–300 ng). (**A**) Western blots display a dose-dependent increase in total, as well as phosphorylated STAT3; (**B**) Quantification of STAT3 phosphorylation; (**C**) Quantification of total STAT3 protein expression. Data were subjected to ANOVA followed by Tukey‘s post hoc comparison test (*n =* 3). * *p* < 0.05 vs. control. Error bars, mean ± S.E. C, control; TR, transfection reagent.

**Figure 7:**
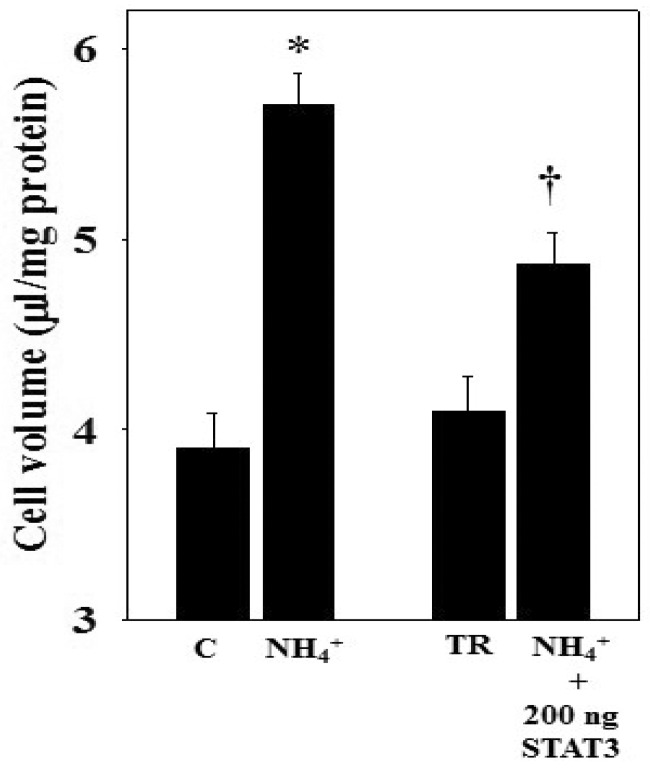
Effect of STAT3 overexpression on the ammonia-induced astrocyte swelling. STAT3-overexpressing cells showed less swelling following ammonia treatment (24 h), as compared to non-transfected cells (WT cells). Data were subjected to ANOVA followed by Tukey‘s post hoc comparison test (*n =* 3). * *p* < 0.05 vs. control and TR; † *p* < 0.05 vs. TR and NH_4_^+^. Error bars, mean ± S.E. C, control; TR, transfection reagent.

Astrocytes were not starved prior to experimentation and the culture media were changed regularly. Additionally, no difference in STAT3 phosphorylation status was observed in plus or minus db-cAMP-treated astrocytes. Moreover, we found no change in basal levels of phosphorylated and non-phosphorylated STAT3, as well as receptor-type protein tyrosine phosphatase (PTPRT) for up to five days. Controls chosen were from 24 h (Figure 2, Figure 3, Figure 4, Figure 5, Figure 6 and Figure 7) and 72 h (Figure 1).

### 3.2. Effect of Ammonia on STAT3 Protein Levels in Astrocytes

Cultures were exposed to ammonia (5 mM) for different time periods (0–24 h) and total STAT3 protein levels were measured by WB. Cultures exposed to ammonia displayed no change in total protein levels for up to 6 h. However, a marked decrease in total STAT3 protein was observed at 12 and 24 h after ammonia exposure (Figure 2).

### 3.3. Effect of Ammonia on Protein Tyrosine Phosphatase Receptor Type-1 (PTPRT-1) Protein Expression in Astrocytes

Cultured astrocytes were exposed to ammonia (5 mM) for different time periods (0–24 h) and the protein expression of PTPRT-1 was measured by WB. Phosphatases such as protein tyrosine phosphatase 1B (PTP1B), Src homology phosphatases (SHPs), protein phosphatase-2 (PP2A), PTPRT-1 and phosphatase and tensin homolog (PTEN), are all well-known to dephosphorylate serine or tyrosine residues of STAT3 [30,31,32,33]. Among these phosphatases, the activation of PTPRT-1 was shown to specifically dephosphorylate STAT3-Tyr705 [34].

Cultures exposed to ammonia displayed a significant increase in PTPRT-1 at all-time points examined (Figure 3), which corresponded well with the decreased levels of p-STAT3 observed following ammonia treatment.

### 3.4. Effect of Sodium Orthovanadate (SOV) and RK-682 on Ammonia-Induced STAT3 Dephosphorylation and Astrocyte Swelling

To determine whether the inhibition of protein tyrosine phosphatase reverses the ammonia-induced STAT3 dephosphorylation (inactivation), cells were exposed to 25 and 50 μM of the general protein phosphatase inhibitor, sodium orthovanadate (SOV), or with the specific protein-tyrosine phosphatase inhibitor RK-682 (25 μM) [35], with and without ammonia for 24 h, and the extent of STAT3 phosphorylation was measured by Western blots. Cultures exposed to ammonia (5 mM) for 24 h showed a decline in STAT3-Tyr705 phosphorylation (Figure 1), while 25 and 50 μM SOV diminished such effect (57.6% and 48.4%, respectively) (Figure 4A). Similarly, RK-682 (10 and 25 μM) significantly reduced the decline in ammonia-induced STAT3 phosphorylation (42.6% and 58.1%, respectively) (Figure 4B).

We next examined whether the inhibition of protein tyrosine phosphatase reverses the ammonia-induced cell swelling in cultured astrocytes. Cultures exposed to ammonia (5 mM) for 24 h showed 46.7% increase in cell swelling as measured by the O-methyl glucose (OMG) uptake method. Pretreatment (15 min) of cells with 50 μM SOV, or with the specific protein phosphatase inhibitor RK-682 (25 μM), diminished the ammonia-induced cell swelling by 64.6% and 52.2%, respectively (Figure 5). While the phosphatase inhibitors SOV (50 μM) and RK-682 (25 μM) alone inhibited STAT3 phosphorylation by 14.1% and 9.4%, respectively, the effects were not statistically significant different from control.

### 3.5. Ammonia-Induced STAT3 Dephosphorylation and Cell Swelling in STAT3 Over-Expressed Astrocytes

Since ammonia decreases STAT3 phosphorylation, as well as total STAT3 protein level, we examined whether the over-expression of STAT3 reverses the ammonia-induced STAT3 dephosphorylation and protein expression, as well as the decrease in cell swelling in astrocyte cultures. Astrocytes were transfected with pExpress1-STAT3 (100, 200 and 300 ng/2.5 × 10^5^ cells) for 48 h, and at the end of transfection the cells were replaced with normal cell media. After 24 h, total and phosphorylated STAT3 were measured by Western blots. Cultures transfected with 200 and 300 ng pExpress1-STAT3 showed a 3–4-fold increase in total and phosphorylated STAT3 (Figure 6). Treatment with the transfection reagent (Mirus TransIT-TKO reagent) (10–20 μL/2.5 × 10^5^ cells) alone did not display any evidence of cytotoxicity.

We next sought evidence as to whether the overexpression of STAT3 influenced cell swelling. Cultures were transfected with 200 ng pExpress1-STAT3 for 48 h, and at the end of transfection, cells were replaced with regular media along with ammonia for 24 h, and the level of cell swelling was determined. Ammonia significantly increased cell swelling in cells that did not receive pExpress1-STAT3. In contrast, pExpress1-STAT3 transfected cells exhibited a reduction in cell swelling (59.1%) after ammonia treatment (Figure 7). Additionally, a 152% increase in STAT3 phosphorylation was identified in ammonia-treated astrocytes after STAT3 overexpression (data not shown), as compared to a 3.5-fold increase in p-STAT3 in STAT3 overexpressed cells without ammonia exposure (Figure 6). While STAT3 overexpression significantly reduced the ammonia-induced astrocyte swelling, STAT3 overexpression alone had no significant effect on cell swelling.

## 4. Discussion

Our study demonstrates that cultured astrocytes exposed to ammonia caused a significant decrease in STAT3 protein expression and phosphorylation. Additionally, exposure of astrocytes to inhibitors of protein tyrosine phosphatases (sodium orthovanadate or RK-682), which are known to enhance the phosphorylation of STAT3, diminished the ammonia-induced astrocyte swelling. Overexpressing STAT3 in cultured astrocytes similarly caused a reduction in the astrocyte swelling induced by ammonia. These findings strongly suggest that dephosphorylation (inactivation) of STAT3 represent a critical event in the mechanism of ammonia-induced astrocyte swelling, and likely in the brain edema associated with ALF.

STAT proteins are latent cytoplasmic transcription factors consisting of seven members (STAT1-4, 5a and b and STAT6 [36,37,38], all of which can be activated by a variety of signaling systems (e.g., MAPKs, epidermal growth factor), as well as cytokines. STAT3 is tyrosine phosphorylated by receptor tyrosine kinases, including EGFR, FGFR and the JAK family of kinases. Phosphatases such as protein tyrosine phosphatase 1B (PTP1B), Src homology phosphatases (SHPs), protein phosphatase-2 (PP2A), receptor-type protein tyrosine phosphatase (PTPRT-1) and phosphatase and tensin homolog (PTEN) are well-known to dephosphorylate serine or tyrosine residues of STAT3 [30,31,32,33]. Among these phosphatases, the activation of PTPRT-1 was shown to specifically dephosphorylate STAT3-Tyr705 [34].

Once activated by phosphorylation at the Tyr705 residue, STAT3 becomes dimerized and translocates to the nucleus where it binds to the DNA [37,38,39], resulting in the activation of many genes involved in the maintenance of cell integrity [40,41,42].

Activation of STAT3 was shown to protect cells from oxidative stress by upregulating the antioxidant enzyme in vitro [14] and in vivo [15]. Alternatively, STAT3 deletion was shown to promote oxidative stress [16], and to activate factors known to induce cell swelling in cultured astrocytes [6,17]. Additionally, increased phosphorylation (activation) of STAT3 was shown to inhibit the activation of NF-κB and inducible nitric oxide synthase gene expression, both well-known factors involved in the mechanism of astrocyte swelling [4,5]. These findings collectively suggest that decreased STAT3 phosphorylation by ammonia may have enhanced oxidative stress, and activation of signaling systems MAPK, P53 and NF-κB that subsequently contributes to the astrocyte swelling [4,5,6].

Phosphorylated STAT3 was observed in brain following intracerebral hemorrhage, a neurological condition associated with brain edema [19]. We now show that STAT3 was dephosphorylated when astrocyte cultures were exposed to a pathophysiological concentration of ammonia. Additionally, treatment of astrocytes with the protein phosphatase inhibitor, sodium orthovanadate or RK-682, significantly diminished STAT3 dephosphorylation, as well as reduced the cell swelling induced by ammonia. Further, astrocytes transfected with pExpress1-STAT3 increased total, as well as phosphorylated STAT3 protein expression, and such treatment was also associated with a reduction in the ammonia-induced cell swelling, suggesting that deactivation of STAT3 represents a critical early event in the process leading to the astrocyte swelling in ALF. While the means by which ammonia activates the PTPRT is unclear, it is possible that ammonia may have activated signaling systems that are known to phosphorylate and activate PTPRT, such as epidermal growth factor receptor, leptin R and avb3 [43].

While increased STAT3 phosphorylation was implicated in the mechanism of cell proliferation in CNS neoplasms [8,9,10,11,12,13], it is possible that decreased STAT3 phosphorylation, which was observed following exposure of astrocytes to ammonia, may have contributed to the impaired astrocyte proliferation following ammonia exposure, likely as a consequence of the increased senescence-associated genes expression levels [44].

## 5. Conclusions

Our studies document that STAT3 is dephosphorylated when astrocyte cultures are exposed to a pathophysiological concentration of ammonia, and that treatment of astrocytes with the protein phosphatase inhibitor, sodium orthovanadate, significantly diminished STAT3 dephosphorylation, as well as the cell swelling induced by ammonia. Further, astrocytes transfected with pExpress1-STAT3 increased total, as well as phosphorylated STAT3 protein expression, and that such treatment was associated with a marked reduction in the ammonia-induced cell swelling. In aggregate, our findings suggest that targeting STAT3 may represent a promising approach for the treatment of the brain edema associated with acute liver failure.
